# A survey and critical analysis of the teaching of medical ethics in UK medical schools

**DOI:** 10.1007/s40889-022-00158-2

**Published:** 2022-11-03

**Authors:** Jan Deckers

**Affiliations:** School of Medicine, Newcastle University, NE2 4HH Newcastle, United Kingdom

**Keywords:** Bioethics, Medical ethics, Medicine

## Abstract

This article surveys and analyses the reflections on medical ethics teaching by colleagues teaching in United Kingdom (UK) medical schools in the early 2020s. Participants were recruited mainly by using the worldwide web to identify 64 people from 41 UK medical schools who were thought to contribute to teaching medical ethics based on their internet profiles. Twenty-three people responded. The survey data reveals that many staff are happy with the provision of medical ethics teaching, but also that some are concerned about the quality of provision due to concerns with staff expertise and teaching time. In spite of the fact that the General Medical Council (GMC) and other organisations are perceived to have contributed to raising the profile of medical ethics, there is significant concern with how it is embedded within local UK medical curricula. Some participants contributed hardly or not at all to research in medical ethics, where one attributed this decline in research to the pandemic. Future work will need to address what can be done to improve the provision of medical ethics teaching to address some of these findings and to survey and analyse how perceptions might have changed in light of recent challenges and developments.

## Introduction

Previous research explored the views held by curriculum leaders and ethics leaders in 28 medical schools in the UK with regard to the nature, methods, opportunities, and challenges related to teaching medical ethics in undergraduate medical curricula (Mattick and Bligh 2006). Whilst 22 schools responded, this data is quite old, dating from 2004. More recent research was conducted in 2015 to survey the views of “those responsible for ethics education at the UK medical schools”, then 34 in number, by contacting “the lead for medical ethics at each medical school”, with the aim to evaluate and to identify barriers to the teaching and assessment of medical ethics, but the number of participants (11) was low (Brooks and Bell 2017, p. 606).

This study initiated in January 2021 and concerns a similar research project on the teaching of medical ethics in UK medical schools, the number of which had now grown to 42. The aim of the project is to survey and analyse the views of those who contribute to the teaching of medical ethics in UK medical schools with the aim to develop a better understanding of current strengths and weaknesses, as well as what might be done to improve the teaching of medical ethics in the UK, and potentially also elsewhere. It is distinct from these earlier studies in a number of respects. Firstly, neither of the two studies just mentioned described the method by which participants were recruited. This is nevertheless important as answers to survey questions can vary significantly depending on who is invited to participate. It is therefore important to set out clearly how potential participants are identified. Secondly, both earlier studies asked participants for personal details, including names and email addresses, which may affect responses significantly due to privacy concerns. No personal details are asked for in the survey that forms the basis for this article. Thirdly, this study includes a shorter lists of questions that includes more open questions to prevent participant fatigue and to encourage greater elaboration in order to stimulate a better response rate and the production of qualitatively richer data. The aim of this article is to report and discuss the findings of this survey. This research is timely as it is important to explore how the recent expansion in the number of UK medical schools and in medical students is being experienced by medical ethics teachers, particularly as this rapid increase is taking place during a time when health care systems, and therefore medical schools, are under unprecedented strain, partly as a result of the SARS-CoV2 pandemic.

## Methods

A study protocol was designed and approval from Newcastle University’s Research Ethics Committee was sought and granted on this basis. Participants were recruited by using the worldwide web to identify all medical schools in the UK. The same web was then used to scan the home pages of these schools in order to try to identify staff working in medical ethics and law. Existing contacts with members in some of these schools were also used, as well as Google searches using the search terms “(name of medical school), ethics, law”. In this way it was possible to identify the email addresses of at least one member of staff working in the area of medical ethics and law within all bar one of the UK’s medical schools. Emails were sent to these, and participants were also asked to pass on contact details of any of their colleagues who either contributed or had contributed to the teaching of medical ethics in the last 2 years. The remit was limited to those with teaching responsibilities. The survey (with May 2021 deadline) was sent to 64 contacts in all UK universities with medical schools, excluding contacts in my own University and in one University where no contact person could be found. Not all of these contacts may have been relevant as some might have had little or no input in teaching on the medical curriculum. However, from personal knowledge and from an analysis of web pages, at least 70% would have had significant input.

Survey questions were developed by means of reflecting on questions asked in earlier, similar surveys (Mattick and Bligh 2006; Brooks and Bell 2017). The list of questions was deliberately kept relatively short to reduce the risk of participant fatigue and out of a preference for a less didactic approach compared to earlier studies. The selection was biased in favour of the overaching intention to find out what those who teach medical ethics in UK medical schools think about current medical ethics teaching and about what the ideal medical ethics teacher and teaching in UK medical schools should look like. Whilst it might have been interesting to collect personal and demographic data on participants, no personal or demographic data were collected to preserve anonymity and because the number of participants was considered to be too low to allow meaningful comparisons on the basis of sex, gender, age, years of experience, or any other ways in which participants might have been categorised. Preserving participants’ anonymity was considered to be paramount to encourage the free flow of ideas and to reduce the risk of participants experiencing negative consequences associated with their exercising freedom of speech. Table 1 lists the questions that participants were asked to answer.

**Table 1:** Questions asked from participants in survey on teaching of medical ethics in UK medical schools

1. How long has medical ethics been taught in your institution?
2. How many people are involved in the teaching of medical ethics in your institution?
3. What did you study?
4. Do you think that those who teach medical ethics in your institution are qualified to teach it? Why/why not?
5. What do you think that your colleagues’ perceptions might be of the relevance of medical ethics? Are you feeling supported by them? Why/why not?
6. Are you researching in medical ethics? Why/why not?
7. Do you think that those who teach medical ethics should also research in medical ethics? Why/why not?
8. Are you involved in any professional organisations related to medical ethics? If any, what are these?
9. What, if anything, do you gain from being a member of the professional organisation(s) that you are a member of?
10. Are you, or have you been involved in peer-reviewing in medical ethics? If so, could you describe your experience?
11. Do you have, or have you had, any editorial responsibilities in relation to medical ethics? If so, could you describe your experience?
12. Do you have, or have you had any duties related to assessing research applications in relation to medical ethics? If so, could you describe your experience?
13. Do you think it is important for medical students to study ethics? Why/why not?
14. Are you satisfied with the amount of ethics teaching that medical students receive? Why/why not?
15. Are you satisfied with the quality of teaching in medical ethics? Why/why not?
16. How is medical ethics being assessed?
17. Are you satisfied with the ways in which medical ethics is assessed? Why/why not?
18. Do you have sufficient opportunities to drive curricular change related to medical ethics? Why/why not?
19. Is there anything else that you think may be relevant for this research?

Responses to these questions were analysed thematically (Corbin and Strauss 2008). Some quantitative aspects have been provided to show how many participants out of the 23 respondents raised particular themes. However, the small number of respondents and the qualitative nature of the project precludes further statistical analysis. Where illustrative of particular themes, quotations have been included. All quotations of participants’ answers are verbatim, with inclusion of linguistic errors.

## Results

Twenty-three people responded to the survey, including one person who had recently left their post, yielding a rich data set. Data were analysed by going systematically through the numbered list of Table 1, as follows:

1. How long has medical ethics been taught in your institution?

Thirteen respondents answered that medical ethics had been taught for over two decades. Six were not sure, including two who thought it had been taught for more than 20 years. Two participants who identified that their medical school had been established in recent years pointed out that it had been taught from the outset.

2. How many people are involved in the teaching of medical ethics in your institution?

One identified “a core management team of 8–10 and other tutors–up to 20”. However, nine respondents identifed that one to four core people were involved. However, many stated that many others were involved as well, including many who were contributing on an honorary or voluntary basis. Two respondents stated that less than a full-time post was devoted to it, where one stated that provision was also from “several clinicians who volunteer their time” and another referred to “assistance from colleagues (clinical) as and when”.

3. What did you study?

There was a lot of variety as to what participants had studied. Six respondents stated that they had obtained a PhD in ethics or philosophy. References were made here to philosophy, empirical ethics, bioethics, and “mental health ethics & law”. Another respondent stated that they had obtained a PhD in a “psychiatry department”. Six identified (also) that they had acquired MA degrees in medical ethics and law, and another respondent just wrote “medical ethics and law”. Ten either referred to medicine as their first qualification or listed it first, and seven mentioned philosophy, psychiatry, or ethics and law as studies in which they had obtained additional qualifications.

4. Do you think that those who teach medical ethics in your institution are qualified to teach it? Why/why not?

Overall, most answered this question positively. However, five respondents expressed some concern that some staff may not be well-qualified, with one writing about there being a “lack of depth and understanding”. Three suggested that the teaching of medical ethics may require both clinical expertise and academic expertise in ethics, but that few possess skills in both areas. One answered, for example, as follows: “Yes, on the whole. I think there is some insecurity about that (certainly on my part, as I have no formal qualification in ethics). However on the whole I think clinical experience is actually better than an academic qualification, if you had to choose between the two. Theoretical knowledge you can acquire from reading books and journals; whereas having to make very complex decisions in challenging environments and then coping with the ramifications for patients and families is an experience which non-medics will struggle to understand or convey. That said, ideally a medical ethics lecturer would have both clinical experience and a postgraduate qualification in ethics; once I finish my masters in medical education I will start one in bioethics.”

5. What do you think that your colleagues’ perceptions might be of the relevance of medical ethics? Are you feeling supported by them? Why/why not?

One spoke of “very good support”, particularly from “practising clinicians”, but also stated that there was “less understanding of the role of ethics teaching in terms of generalisable skills such as normative reasoning and structuring a discussion”. A similar comment was made by another who spoke of people being “generally supportive”, but “most” having “limited understanding of our work”. A third respondent also spoke about being supported well, but about some thinking that “it is a fluffy subject with no right or wrong answers and is just theoretical navel-gazing”. This perception of a lack of understanding was specified by six participants. One wrote that some regard it as a “woolly subject” and that “not all educators are comfortable with ambiguity”. Another wrote that “I think that we are supported by colleagues but viewed as either being a) somewhat mysterious or b) teaching the ‘bleeding obvious’.”

One respondent related this perception of support to the GMC, writing that support had “only increased over time as the GMC has formalised ethics and law learning outcome”. This perception that medical ethics was important might also have stemmed from a reflection upon the significance of having adequate numbers of staff, where the same person wrote for the second question that 5 people, as well as doctoral students, were involved. Interestingly, another respondent who identified 2.7 permanent staff and over 20 in total being involved, wrote similarly that it was “generally held in high regard”.

The most detailed response to this question came from one respondent who wrote that some do not understand how relevant it is, writing: “The curriculum remains filled with bio-medical science, the facts of which students are required to learn by rote, and yet most of this information is available on the internet. Some of the information, particularly the therapeutic regimes, will be out-of-date in 10 years time. The communication and analytic skills developed in the ethics course will serve these future doctors for life.”

6. Are you researching in medical ethics? Why/why not?

Nine respondents stated that they did not do any research in medical ethics, and another five responded that they did very little. Thirteen attributed the fact that they did no or hardly any research to the nature of their contract and/or to a lack of time. One wrote: “there is nothing currently presenting itself to me that I have the skills to research”. Another wrote: “I am on a teaching-focussed contract so in theory my time is split 80/20 between teaching and research. In reality I get way less time than that for research because we are too badly understaffed … teaching is always so pressed.” This lack of time was related to the pandemic by one, who wrote: “COVID has derailed things, but I do still do research … there are still times when I just want to be doing more research”. Only one respondent claimed to be spending a lot of time on research, where they wrote: “I am funded by [the] Wellcome [Trust] to do so 60% of my time and I love it!”

7. Do you think that those who teach medical ethics should also research in medical ethics? Why/why not?

Six thought that the answer should be positive. One wrote: “definitely”. Another elaborated: “Research makes you very aware of the intricacies of the problem … makes one more passionate about the topics one teaches”. One contrasted doing research with being a “medical educator”, which they opposed to their academic ideal.

Four participants responded to this question that the answer should be positive in the ideal world, but that this may not be possible in reality, due to time constraints. One wrote: “many medical ethics departments are on the small side, and therefore there is an awful lot of work spread between few hands.” Another wrote: “I would love to do more research in medical ethics but I am a part time member of staff with a heavy teaching workload.”

Around half perceived research – to a greater or lesser degree – to be an optional extra, with two perceiving it to be “a bonus” and another writing that it is desirable to have some researchers within a teaching team. Some expressed that some people are more suited to research and others more to teaching.

One contrasted research with scholarship: “If not research at least be on top of scholarship and developments.” Three questioned the benefit of researching. One wrote: “Being a good teacher has nothing to do with research. Undergraduate medical school teaching requires a broad understanding of clinical ethical issues.” Another wrote that researchers “have a tendancy to become somewhat out of touch with the realities of practising medicine”.

8. Are you involved in any professional organisations related to medical ethics? If any, what are these?

Ten respondents declared to be members of the Institute of Medical Ethics (IME) and ten responded no. The UK Clinical Ethics Network (UKCEN) was mentioned twice. Other organisations that were mentioned included: General Medical Council, Royal College of Physicians of London, Faculty of Public Health, Australasian Faculty of Public Health, the Health and Care Professions Council, and the UK Forum for Professionalism.

9. What, if anything, do you gain from being a member of the professional organisation(s) that you are a member of?

Things that people reported to gain from professional organisations included opportunities for networking, sharing experiences, and curriculum development, being provided with information about conferences and lectures, opportunities for quality assurance and feedback, and supporting medical students and junior doctors in medical ethics. Loftier claims included “the ongoing evolution of the field of medical ethics in the UK … and the potential to pioneer new educational activities and research”, “raising profile and reach of medical ethics in the UK”.

10. Are you, or have you been involved in peer-reviewing in medical ethics? If so, could you describe your experience?

Eight wrote that they did not do any peer-review. One wrote: “I peer review for lots of journals - not huge amounts, but maybe 10 papers a year. I enjoy it and obviously also appreciate the importance of it: I get to read a lot of interesting papers, keep my own critical skills fairly sharp, and feel like I’m pulling my weight in terms of the academic community.” Another wrote: “I take reviewing to be an important reciprocal obligation for those scholars who are applying for grants or who are dependent on peer reviewers for the publication of their own papers.” Five wrote statements that showed that they had limited experience, with one writing: “I do think it’s important. I appreciate journals that give the reviewer feedback.” Whilst most understood the question to be about peer-reviewing for journals, one referred to their being a line manager for “module clinical advisers in medial ethics and law” and another wrote: “Only informal peer reviewing for full-time academic colleagues.”

11. Do you have, or have you had, any editorial responsibilities in relation to medical ethics? If so, could you describe your experience?

The vast majority of respondents had little to no experience with this. Eighteen respondents had no experience related to editorial work related to medical ethics. Seven had been involved in some editorial work, even if not necessarily related to medical ethics. One had been an associate editor for the Journal of Medical Ethics and another had some role with the publisher of the same journal, even if not within the editorial board. One saw “editorial responsibilities as vitally important in stewarding the direction of travel for research in the field of medical ethics”.

12. Do you have, or have you had any duties related to assessing research applications in relation to medical ethics? If so, could you describe your experience?

Fifteen provided a negative response here. One referred to reviewing grant applications, but none related to medical ethics. Another referred to reviewing “grant applications for national and international charitable foundations and for government funding schemes”, which they claimed had become more time-consuming. Four claimed to be members of Research Ethics Committees, where three specified this to be a committee within their University. Of this work, one claimed that it was “under-resourced and under-recognised”.

13. Do you think it is important for medical students to study ethics? Why/why not?

Rich accounts were provided in response to these questions, both quantitatively and qualitatively. The themes that emerged include the following things repondents thought medical students should learn from studying ethics: a/ to develop generic analytical skills; b/ to prepare for ethical and legal clinical and research practice; and c/ to prepare for policy-making in health care.

The first theme emerges clearly from one respondent, who wrote: “Well, I think it’s important for anyone to study ethics. Most of us have a tendency to think sloppily. That’s just natural, I think. But ethics can a) show us why something is a problem b) show us the different aspects of the problem and c) give us a sense of the complexity and reasons why we do what we do. Ethical problems are part of daily life. In the medical field, that is very obvious. Training students into reflecting and analysing the issues they encounter (including the fact that some situations are morally problematic, although they did not see them that way) is essential to help them make choices that they are able to justify to themselves and others.”

The second theme is represented by the respondent who wrote: “They need to recognise the ethical component of clinical encounters and have the skills to reason these so that decisions and choices are well made and less likely to be subject to prejudice and bias”. A more elaborate account of the same sort of idea was provided by another respondent, as follows: “Yes. They need to know the ethical/legal principles governing medical practice so as to understand what is required/permissible and appropriate/desirable. Medical Ethics & Law teaching will equip them to identify, analyse, and deal with, issues that can arise in practice, both in the everyday clinical encounter and, more broadly, in medical science and healthcare practice. The knowledge and understanding gained will form the foundations for good quality and confident decision-making in practice.”

Whilst neither of these two respondents related their answers to what the goal of medicine might be, one went further by doing so, writing as follows: “The primary function of medicine is to my mind an ethical one - the relief of human suffering and promotion of human wellbeing. Medicine is perhaps the most ethically challenging of all the professions, and the safety of patients and the wellbeing of doctors both mandate a thorough understanding of ethical concepts and perspectives which can be used as tools to navigate a rapidly changing clinical environment. Moral reasoning is as important as clinical reasoning for any healthcare professional. Even at undergraduate level, students will need a firm grounding in ethics to identify and combat problematic elements of the hidden curriculum and successfully develop an appropriate professional identity. There isn’t really medicine without ethics.” Whilst clinical reasoning and moral reasoning were separated by this respondent, another questioned the juxtaposition of clinical and ethical skills, writing: “I do not separate out clinical skills from the application of ethical skills.”

The final theme came to the fore in the comment of the respondent who wrote: “Some of them will go on to be policy-makers and potentially big movers in that space, so we really need them to be clued up to ethical theory and be able to make sensible, defensible arguments.” Regardless of whether or not medical doctors end up being policy-makers, this wider, holistic, dimension was also identified by another respondent, who wrote: “it is ultimately about looking for ‘wholeness’ in life”.

14. Are you satisfied with the amount of ethics teaching that medical students receive? Why/why not?

About half of the respondents were relatively unhappy with the amount of ethics teaching, with one saying that “we barely scratch the surface”, and another writing that they engaged in “curriculum creep” to try to increase hours. The other half were relatively happy, but explained that they could not do as much small group teaching as they would like. There was some concern that there was relatively little ethics teaching in the later stages of the curriculum.

15. Are you satisfied with the quality of teaching in medical ethics? Why/why not?

People were generally satisfied, but some talked about the challenge of delivering good quality teaching with limited resources, necessitating the need to help other tutors so that they can contribute: “The challenge is delivering small-group discursive learning whilst maintaining quality across the board. I spend a lot of time on staff training and development, producing materials to support facilitators, and quality assurance. Feedback from students suggest that this is successful, but I’m aware that sometimes students “don’t know what they don’t know”. However, I believe that the benefits of learning ethics through discussion outweigh the disadvantages.” This concern about the teaching quality provided by others who were not educated in disciplines that are closely aligned with medical ethics was echoed by another respondent, who wrote: “It is difficult for clinical educators and junior teachers to develop the skills needed to be good teachers of medical ethics and law …”.

16. How is medical ethics being assessed?

Some stated that they did not know whether ethics was being assessed, with one adding “in my institution there is alot of discussion around whether ‘ethics’ should be assessed”. Some reported that there was no separate assessment for ethics, but that it was integrated “with other subjects by portfolio”, “clinical skills”, “formal professionalism judgements”, “reflective writing”, “reflective record”, or “group contributions”. Some identified the use of multiple choice questions (MCQs) and single best answer exams. One idenfied the use of the Applied Knowledge Test (“AKT”), another the use of situational judgments tests (“SJTs”), and a third the use of one best answer questions (“OBA”) and a “week long open book case study”. Some identified the use of short answer questions, with one commenting that this was in response to an “evolving scenario”. Some identified the use of essays, with one commenting that the latter was a final year essay “on a case in their experience”. Some identified the use of objective structured clinical examinations (OSCE’s). One wrote that there was “a distinctive ethics and law OSCE station that sits alongside 12 other clinical stations”. Another wrote about OSCE’s having “ethical and basic legal content”.

It was unclear whether ethics was being assessed throughout the course. If this was the case in any of the relevant institutions, this appeared to be the exception rather than the norm. One contributor wrote that students had the option to answer sociology or ethics questions in year one, and that they would only have one other summative assessment in the final year. Another answered “as part of the general final exam. So, not very well.” One reason relatively little time was devoted to ethics assessment is illustrated beautifully by this respondent, who wrote: “Currently it’s a mixture of multiple choice questions and short answers questions. Some argue for a move to MCQs across the board but I think this is a bit ludicrous - different skills need to be tested in different ways and while it may be easy and neat to test everything in the same way, the realities of a rounded education do not allow for it. I’d like to see more long essay type assignments but we don’t have the capacity to mark them.”

17. Are you satisfied with the ways in which medical ethics is assessed? Why/why not?

Eleven respondents stated that they were satisfied and nine that they were not. Amongst those who were happy, one wrote that they “have MCQs/OBA/Short notes throughout all years … final year OSCE stations and … the open book assessment”. Another claimed to be satisfied, but added: “SAQs are preferable to MCQs because they allow a bit more depth, although there are still some limitations (and they take ages to mark).”

Amongst those who were satisfied as well as those who were dissatisfied, several pointed at the need for what one termed the need for “the university to employ many more members of staff” to assess ethics properly so that it would be easier to identify students who did not have a “true appreciation of the ethical aspects of medical cases”. One respondent who was dissatisfied would like to see “more long essay type assignments but we don”t have the capacity to mark them”. Several identified the use of MCQ’s as inappropriate, even if its validity to assess legal aspects was also recognised.

This lack of satisfaction was also associated by some with the idea that professionalism might not be assessed appropriately. One expressed the idea that the GMC should have a final year ethics exam so that the “presence of the exam would strengthen the status of medical ethics throughout the course”, venting his frustration about the lack thereof as follows: “Other professions have ethics exams, why not doctors? It would boost public confidence in the profession and demonstrate that something is being done after the embarrassments of Bristol, Alder Hey, Mid Staffs, Gosport, Paterson and the issues raised by Cumberledge. In most UK medical schools, students can fail their medical ethics assessments and yet still qualify as doctors, the public would be horrified to know this.”

18. Do you have sufficient opportunities to drive curricular change related to medical ethics? Why/why not?

Views on this question were mixed. Some stated that they felt able to drive such change due to the recognition that was given to external influencers such as the GMC and the Institute of Medical Ethics, for example by referring to being given the opportunity to “align” the curriculum “with the new GMC outcomes for graduates” (GMC 2020) or to using “the IME core curriculum document” as a guideline. This reference to the IME core curriculum document relates to an initiative started by the IME in 1998 where a number of UK teachers of medical ethics agreed on its core content (Consensus statement by teachers of medical ethics and law in UK medical schools 1998). It was revised between 2009 and 2010, resulting in the publication of a new ‘consensus statement’ in 2010 (Stirrat et al. 2010).

There was a great deal of variety in how those who contributed to medical ethics teaching felt empowered by curriculum leaders, with one commenting that “some times I am a lone voice” and another that, “as director of the ethics and law thread course, i am member of the committees that oversee and govern the clinical course”. Some felt that there was a lack of recognition of the importance of medical ethics amongst some colleagues which impaired curricular improvement, with one commenting: “there is still an obsession with teaching medicine as a scientific discipline. Medicine is part-science, but it is also a social profession where empathy, communication skills and resilience are equally important. The GMC, by my reading, have recognised this but it has not filtered through to the curricula of many universities”.

19. Is there anything else that you think may be relevant for this research?

Eight respondents answered the final question. One expressed their hope that this would help to keep medical ethics firmly on the table in medical schools. This was phrased as follows by another: “ethics needs to get better recognition as a core component of medical education”. A similar hope was expressed as follows by another: “everyone involved in teaching at medical schools needs to take a long hard look at the GMCs graphic in Graduate Outcomes and consider how much medical ethics and professionalism is in each section” (GMC 2020). Another expressed the hope for more “professional support” and to “get medical ethics teachers together to work on specific issues-this can guide research”.

One participant apologised for giving short answers, adding that “this is fantastic research, but time pressure is all a bit too much at the moment”. One expressed the desire to open up medical ethics as follows: “the biosphere needs support from us human beings. Environmental and Development Ethics are essential perspectives that need to be addressed. Medical ethics should not sit in a silo. It should be about the world as a whole. Health includes everything, that is why it is so difficult. The Ayurveda/Yoga work in Sanskrit for health is ‘svastya’ - being rooted within, and that is what we should be teaching in medical ethics. How can each of us be ‘rooted within’ particularly if we are taking on the role of a medical practitioner.”

## Discussion

Medical ethics appears to be well-established in UK medical schools as respondents identified that it had been taught for a long time or, in relatively new medical schools, from the outset. The number of staff involved in its delivery, however, is highly variable. Whilst this variation can be attributed to some extent to student intake being variable, there is no question that some institutions attach much greater importance to medical ethics compared to others. Responses varied from “a core management team of 8–10” to less than a full-time post. Whilst it is encouraging to read that some schools benefit from voluntary contributions, this is also of some concern due to the possibility that some voluntary contributors may contribute on a voluntary basis merely because they are not provided with the opportunity to be remunerated. As most human beings are moral agents, but most human beings are not anatomists, oncologists, etc., this tendency to hire voluntary contributors may stem from a perception that ethics, unlike other subjects, can be taught by virtually anyone who is a moral agent.

This perception may also account, to some extent, for why contributors identified a wide range of different studies that they had undertaken. Such variation may be positive in that clinical decision-making is complex. This complexity must be understood against the background of the complex human being in the complex clinical setting, necessating input from disciplines including psychology, sociology, law, and health technology. However, none of these disciplines define the field of moral philosophy. Therefore, whilst contributions from other disciplines must be valued, they should never be traded off with the discipline of moral philosophy or ethics, which integrates elements from other disciples insofar that they are relevant to make moral decisions.

Whilst most respondents thought that those who teach medical ethics in their institutions are qualified to teach it, there was some concern that some staff may not be well-qualified. This might be interpreted to stem from such trade-offs. Whilst it might be countered that moral philosophers are not adept at understanding how clinical complexities ought to affect moral decision-making, this, in my view, does not yield an argument that moral philosophy should be supplemented by other approaches. Rather, it is an argument that moral philosophers should be better at doing moral philosophy. This does not imply that those without any formal qualification in ethics should necessarily be barred from teaching medical ethics. Whilst one respondent may have regretted not having a formal qualification, being skilled to teach medical ethics is not tantamount to having a degree in it.

With one respondent, we might agree that having clinical experience is better than having an academic qualification, but we should always be mindful of the fact that there is no such thing as a bare experience that we are exposed to without the bias from our interpretative frameworks (Mohanty 2008). Philosophy is about understanding these frameworks so that we can make moral decisions in light of rationales that we no longer adopt uncritically. If students are never subjected to what one referred to as “theoretical navel-gazing”, the potential for students to become entrapped into problematic theories that are never questioned is bound to be greater. The benefit of “theoretical navel-gazing” is that it allows students to explore alternative options that may be either accepted or discarded. The idea that it is meaningless to contemplate alternative options that are “off the wall” and that will be discarded anyway loses force when one keeps an open mind about alternative options and when one realises that a good justification of one’s own ethics depends on one’s ability to provide a reasoned account as to why alternative ethics should be rejected (Jamieson 1991).

This process is painful. It is much easier to take some values for granted and to derive some logical outcomes from these. Some participants identified that much medical education does precisely that. Students need to know some facts that are widely accepted to be relevant to their health care practices. Even if some of these facts may be disputable, many will be widely accepted to be indisputable. An example is the idea that one should not treat a virus with an antibiotic. There may no longer be any need to question this. As many facts about medicine are just about beyond dispute, some staff may be relatively impatient with medical ethics. This comes through in the comments from those who wrote that it is perceived by others as a “woolly subject” or that it is “somewhat mysterious” where it is “bleeding(ly) obvious”. One might add to one comment that, regardless of whether or not facts will still be held to be facts in ten years time, “the communication and analytic skills developed in the ethics course will serve … future doctors for life” not only because they will help them to deal with accepted facts, but also to determine which facts should (continue to) be accepted as facts.

Whilst some colleagues were perceived not to appreciate the relevance of medical ethics sufficiently, it was good to read that some felt that the formalisation of some ethics curriculum by the GMC had helped to garner support. Further support might be provided by the use of academic resources that may not only help students, but also the many non-specialists who contribute to teaching the subject. Whilst participants mentioned GMC documents, some medical ethics journals, and the four principles approach (Beauchamp and Childress 2001), none mentioned specific textbooks, suggesting that existing textbooks were not used widely or that there might be a dearth of appropriate resources.

It is worrying to read that the majority of respondents stated that they did not research in medical ethics, that hardly anyone had any editorial experience, and that several expressed some frustration with not being able to do (more) research. Many attributed this to the nature of their contract and/or to a lack of time. One related this lack of time to COVID-19 having “derailed things”. As this survey was closed just after the start of the pandemic, this problem might plausibly have been aggravated. However, one also referred to a lack of skill. Only one respondent claimed to be spending a lot of time on research, which was facilitated by external funding. Whilst I do not adopt the view that those who contribute to teaching medical ethics must necessarily engage in research, the analytical skills that are so crucial for the discipline are honed by research. The maintenance and development of these skills cannot be taken for granted. Therefore, it seems desirable for teachers also to be active in research. Whereas research skills can be maintained and developed in other ways, I am rather concerned by the growing trend in UK higher education to divide academic staff between researchers and teachers (Cotton et al. 2018; McKinley et al. 2021). This concern appeared to be shared by at least one respondent, who contrasted doing research with being a “medical educator”, which they opposed to their academic ideal. I am also concerned about the fact that one respondent separated research from scholarship, suggesting that those who teach medical ethics should “at least be on top of scholarship”. Whilst I do not claim that scholarship and research are identical, I am concerned that the former might be used to justify the denial of research time for those who are on contracts that focus heavily on teaching. Whereas universities in the UK would historically demand that staff are active in teaching and research, a relatively new trend in some institutions is that some staff are offered “teaching and scholarship” contracts that may serve to diminish the importance of research for some staff. Nevertheless, I also share the concern of one respondent who stated that researchers might “become somewhat out of touch with the realities of practising medicine”.

It is somewhat concerning that about half of the respondents were not members of any professional organisations related to medical ethics, particularly as some thought that being a member of such an organisation might help to raise the profile of medical ethics. Some might not seek membership as they may believe that there may be no need to try to raise the profile of this discipline or that professional organisations should not be used to do so. This interpretation, however, may be unlikely to be valid in light of the fact that several expressed concern over the profile of medical ethics, both internally and externally. For professional organisations, it is paramount to explore to what extent some people may not be able to become members of such organisations and of the conferences and journals that they may host because of some people’s inabilities to pay memberships, conference registrations, and publication charges related to open access publication, and for these inequalities to be addressed where they cannot be justified.

Participants’ concerns about the profile of medical ethics might also account, to some extent, for why some thought that curricula might not value medical ethics sufficiently, particularly in the later stages, and for why some expressed some dissatisfaction with the quality of teaching in medical ethics. This dissatisfaction was related by some to the fact that there was a lack of specialised staff, necessitating significant involvement of non-specialist staff, where some recognised the need to trade off good tutor input by means of less appropriate large group lectures versus worse tutor input by means of small group sessions, which are widely held to be more appropriate for ethics teaching (Giubilini et al. 2016). It is very worrying that a similar recent study on UK undergraduate medical curricula also identified “a reduction in the number of schools with full-time academics taking responsibility for ethics education since 2006” during a time when student numbers have increased very significantly, which is why the authors argued that “there is a need to address why there are so few full-time academics leading medical ethics teaching” (Brooks and Bell 2017, p. 612). Whilst several respondents valued working as members of Research Ethics Committees, and presumably were valued for doing so, this concern about the profile of medical ethics might also be implicit in the comment of one respondent who wrote that this kind of work was “under-resourced and under-recognised”.

I agree with participants about the importance of peer-review, and with the reasons participants gave. I am a bit concerned about the comment from one respondent who wrote that they “appreciate journals that give the reviewer feedback”. I believe that journals should provide good feedback, and I wonder whether this comment might be based on the experience that some journals do not provide any feedback at all. If scholars in medical ethics might feel more strongly that they are part of a scholarly community, which might be cultivated by some professional organisations, the probability of some reviewers not taking their “reciprocal” responsibilities seriously might diminish. This is not to say that membership of a professional organisation cannot also be fraught with the danger that it might discriminate unjustifiably against those who are not members of such “communities”.

As mentioned, both quantitatively and qualitatively rich accounts were provided in response to the questions whether and why/why not participants thought it is important for medical students to study ethics. I agree with these accounts. The comment that clinical skills should not be separated from ethical skills is interesting as these were separated by another respondent, perhaps suggesting that some institutions may focus insufficiently on the development of the more ethical rather than the factual accounts of clinical encounters. I found it interesting to read that some respondents associated ethics with politics and policy-making, themes that may not be mentioned frequently in curricular discussions. This comes to the fore in the comments that ethics should “identify and combat problematic elements of the hidden curriculum” (Hafferty and Franks 1994; Goldie 2000; Giubilini et al. 2016), and that ethics should include preparing students for policy-making.

These loftier ambitions appeared somewhat at odds with dominant ways in which ethics was being assessed. Whilst the facts that a diversity of methods was being used and that ethics was integrated with other domains in the way it was being assessed might be embraced, I believe that it is fair to question also whether this diversity and integration might indicate that ethics might not be assessed very well. In terms of its integration, there might be some truth in the claim that, if ethics is being assessed everywhere, it is being assessed nowhere. The difficulties around the assessment of ethics were also foregrounded by the comments that whether ethics should be assessed was an item of significant discussion, and that there was a lack of capacity to mark essays, meaning that assessment by means of essays had to be scrapped. In my view, the author of the latter comment was rightly concerned whether this is compatible with the desire to provide a “rounded education” – an issue that I have commented on elsewhere in light of this concern with marking competency (Deckers 2019). Whereas the use of short answer questions might help to address this issue, and its usefulness was recognised by some respondents, one expressed the concern that marking such questions might still be time-consuming, whilst another made a similar comment about there being a need to employ more staff to assess ethics properly.

This does not imply that all ethics assessment must be onerous. I agree with one participant who wrote that the assessment of the legal aspects of medicine can be done by means of MCQs as students can be rewarded for producing the correct answer in the legal domain. The question of which aspects of ethics can be assessed by means of MCQs depends on what it is that one tries to assess. Whilst this question was not asked directly, I agree with respondents that it must include critical reasoning skills, but also skills that are much more difficult to assess, including whether or not one behaves professionally. I am not sure whether one participant is right to think that “the public would be horrified to know” that students can graduate even if they fail their ethics assessments, but I am very concerned – noting that this concern is shared by others (see e.g.: Mattick and Bligh 2006; Brooks and Bell 2017; Ignatowicz et al. 2022) – that students may qualify in spite of their unprofessional/immoral behaviour. It is clear from the examples that the respondent provided that their concern related less to students’ abilities to demonstrate critical reasoning skills than to their ability to behave appropriately.

It is clear from the above that many respondents thought that they still had to argue the case for “better recognition” of ethics, which also came to the fore in their responses to the questions whether they had sufficient opportunities to drive curricular change and whether there was anything else that participants thought might be relevant for this research. Given that some participants felt supported by external influencers such as the GMC and the IME in their attempts to do so, where one pitched the GMC against many universities’ “obsession with teaching medicine as a scientific discipline”, suggests that external organisations might have an important role to play, raising the question whether there might be institutional and/or financial obstacles as to why many were not members of any such organisations. In relation to the final question, it was encouraging to read the comment that medical ethics should be complemented by other themes, such as themes that feature in ecological ethics. Whilst I think that it cannot really be separated from it as I agree with the comment that “health includes everything”, the need to prepare students for the most common issues that feature in the patient-doctor encounter should, in my view, be used to justify narrowing down the scope of ethics somewhat. However, support for the introduction of broader themes, including themes from ecological ethics, is clearly growing (see e.g. Shaw et al. 2021), and I believe that it is paramount for medical ethicists to be constantly alert to the concern of curricular constriction.

This study is limited. It is limited as nobody who teaches medical ethics could be identified/contacted in one medical school. It is also limited by the response rate. It is not known whether some did not respond due to a lack of interest or due to the fact that they may not or no longer be involved in teaching. Additionally, whilst contacts were asked to send the survey on to colleagues who were involved in the teaching of medical ethics, where some responded that they had done so, some people will not have received the invitation to contribute. It is also limited in that it remains unclear how this study might contribute to the aim to develop the teaching of medical ethics in the UK. More reflection and dialogue with participants and others is needed to draw out valuable lessons that could be used to develop the discipline, perhaps by the creation of another core document that identifies some pathways along which the discipline might be developed.

## Conclusion

In spite of its limitations, this article presented – to my knowledge – the first account that surveys and analyses the reflections of those who contribute to medical ethics teaching in UK medical schools in the early 2020s on what medical ethics teaching is in UK medical schools today, what it ought to be, and who ought to teach it. As ethics permeates the behaviour of everything that moral agents do, medical ethics is both ordinary in clinical practice and extraordinary in that value reflection may be in constant tension with the need to perfect and routinise clinical practice so that it becomes *second nature*. This may account, to some extent, for the perceptions of those who thought that medical ethics should be embedded more firmly in UK medical curricula and who may perceive that some of their colleagues think it is always there and – to reiterate the words of one respondent – “don’t know what they don’t know”. In spite of this concern, some respondents recognised that statements put out by external organisations, such as the GMC and the IME, might be used to increase local leverage, even if many participants were not members of any professional organisations.

It is concerning that some participants contributed hardly or not at all to research in medical ethics, where some signalled a lack of interest or time. This situation might have aggravated for various reasons, including: the pandemic having created the need to develop new teaching methods and having increased pressure on clinical teaching; increasing financial concerns; and rising student numbers. Future work by medical ethicists will need to address what can be done to improve the provision of medical ethics teaching in light of these findings and to survey and analyse how perceptions from those who work in UK medical schools might have changed in light of recent challenges and developments.
